# Supplementary material for: The Light- and Jasmonic Acid-Induced AaMYB108-like Positive Regulates the Initiation of Glandular Secretory Trichome in Artemisia annua L
Source: Int J Mol Sci. 2023 Aug 18;24(16):12929. doi: 10.3390/ijms241612929 (PMC10455203; doi:10.3390/ijms241612929)
Supplement: Supplementary file 1 [file ijms-24-12929-s001.zip › ijms-2559954-supplementary.pdf]

Supplementary Material

Table S1. Primers used in this work

Primer name	Primer sequence	Experiment
AaMYB108-like-F	GTTCACTATTGCGGCTGTTTTTCT	Cloning
AaMYB108-like-R	TTACATGCTGTTAAAATGTTGTTC	Cloning
AaMYB108-like-RT-F	TCCTATCAGTGAGAGCCAAAACCA	qRT-PCR
AaMYB108-like-RT-R	CCACAAACTGTCCGAAAAATCTCC	qRT-PCR
RT-Actin F	CCAGGCTGTTCAGTCTCTGTAT	qRT-PCR
RT-Actin R	CGCTCGGTAAGGATCTTCATCA	qRT-PCR
1391-proMYB108-like-F	CAGGTCGACGGATCCGAGGTAAGTGACCGCCCAAGAC	GUS staining
1391-proMYB108-like-R	TCAGATCTACCATGGGCACTTTTCTTGATATATAACT	GUS staining
pHB-AaMYB108-like-F	CGGGATCCATGGAACATAAGA	AaMYB108-like overexpression
pHB-AaMYB108-like-R	GGACTAGTCATGCTGTTAAAATGTTG	AaMYB108-like overexpression
AaMYB108-like-Anti-F	GGACTAGTATGGAACATAAGATGACCA	AaMYB108-like antisense
AaMYB108-like-Anti-R	CGGGATCCCATGCTGTTAAA	AaMYB108-like antisense
AaMYB108-like-AD-F	GAGGCCAGTGAATTCATGGAACATAAGATGACCATTA	Y2H
AaMYB108-like-AD-R	GAGCTCGATGGATCCCATGCTGTTAAAATGTTGTTCA	Y2H
AaHD8-BD-F	GAGGCCAGTGAATTCATGTGTCTTGCTATTCTCATGTCATC	Y2H
AaHD8-BD-R	GAGCTCGATGGATCCTTAGTTGTTATCAGACGAAAGTGCC	Y2H
pHB-AaHD8-YFP-F	TCTAAGCTTGGATCCATGAGTTTTGGGGGTTTTCTT	Dual-Luciferase
pHB-AaHD8-YFP-R	GCTCACCATACTAGTGTTGTTATCAGACGAAAGTGCC	Dual-Luciferase
0800-pAaHD1-F	CAGCCCGGGGGATCCAAATCCAAATATAGACCGCTTGA	Dual-Luciferase
0800-pAaHD1-R	CCAAAGCTTCTCGAGCTTTCCTCTACAAAACTCAAAAAAT	Dual-Luciferase
AaHD1-RT-F	GCTTGATCCTCACTGCGGTAT	qRT-PCR
AaHD1-RT-R	TACGTTCTGGGACGAGTTGCT	qRT-PCR
